# ^18^F-Fluorothymidine PET is an early and superior predictor of progression-free survival following chemoimmunotherapy of diffuse large B cell lymphoma: a multicenter study

**DOI:** 10.1007/s00259-021-05353-9

**Published:** 2021-04-28

**Authors:** Ryogo Minamimoto, Luis Fayad, Julie Vose, Jane Meza, Ranjana Advani, Jordan Hankins, Felix Mottaghy, Homer Macapinlac, Alexander Heinzel, Malik E. Juweid, Andrew Quon

**Affiliations:** 1 Division of Nuclear Medicine and Molecular Imaging, Department of Radiology, Stanford University Medical Center, Stanford, CA USA; 2 Division of Nuclear Medicine, National Center for Global Health and Medicine, Tokyo, Japan; 3 Departments of Lymphoma and Myeloma, Division of Cancer Medicine, The University of Texas, MD Anderson Cancer Center, Houston, TX USA; 4 Division of Oncology and Hematology, Department of Internal Medicine, University of Nebraska Medical Center, Omaha, NE USA; 5 Department of Biostatistics, University of Nebraska Medical Center College of Public Health, Omaha, NE USA; 6 Division of Medical Oncology, Department of Internal Medicine, Stanford University Medical Center, Stanford, CA USA; 7 Department of Radiology, University of Nebraska Medical Center, Omaha, NE USA; 8 Departments of Nuclear Medicine and Oncology, Center of Integrated Oncology (CIO), Universities of Aachen, Bonn, Germany; 9 Cologne and Duesseldorf and Comprehensive Diagnostic Center Aachen (CDCA), University Hospital of Aachen, Aachen, Germany; 10 Department of Nuclear Medicine, MD Anderson Cancer Center, Houston, TX USA; 11 Department of Radiology and Nuclear Medicine, University of Jordan, Queen Rania Street, Al Jubeiha, Amman, 11942 Jordan; 12 Division of Nuclear Medicine and Molecular Imaging, Department of Pharmacology, David Geffen School of Medicine at UCLA, Los Angeles, CA USA

**Keywords:** FLT, FDG, DLBCL, PFS, PET/CT

## Abstract

**Purpose:**

To determine whether interim 3′-deoxy-3′-[^18^F]fluorothymidine (iFLT) PET/CT is a superior predictor of progression-free survival (PFS) compared with interim ^18^F-fluorodeoxyglucose (iFDG) PET/CT in patients with diffuse large B cell lymphoma (DLBCL) treated with rituximab, cyclophosphamide, doxorubicin, vincristine, and prednisone (R-CHOP) or rituximab, etoposide, prednisone, vincristine, cyclophosphamide, and doxorubicin (R-EPOCH).

**Methods:**

Ninety-two prospectively enrolled patients with DLBCL underwent both FLT-PET/CT and FDG-PET/CT 18–24 days after two cycles of R-CHOP/R-EPOCH. Deauville-criteria, PERCIST1.0, standardized uptake value (SUV), total lesion glycolysis (TLG), and metabolic tumor volume were used to interpret iFDG-PET/CT while dichotomous visual interpretation was used to interpret iFLT-PET/CT and the results were compared with the 3- and 5-year PFS.

**Results:**

iFLT-PET/CT was negative in 67 (73%) and positive in 25 (27%) patients. iFDG-PET/CT by Deauville criteria was negative (Deauville scores [DS] of 1–3) in 53 (58%) and positive (DS = 4–5) in 39 (42%) patients. Of the 67 iFLT-PET/CT-negative patients, 7 (10.4%) progressed at a median of 14.1 months whereas 14/25 (56.0%) iFLT-PET/CT-positive patients progressed at a median of 7.8 months (*P* < .0001). Of the 53 Deauville-negative patients, 9 (17.0%) progressed at a median of 14.1 months whereas 12/39 (30.8%) Deauville-positive patients progressed at a median of 5.6 months (*P* = .11). In multivariate analysis, including iFLT-PET/CT, PERCIST, interim TLG, and interim SUV_max_, only iFLT-PET/CT was an independent predictor for 3- and 5-year PFS (*P* < .0001 and *P* = .001, respectively).

**Conclusions:**

In patients with DLBCL given R-CHOP/R-EPOCH, iFLT-PET/CT is a superior independent predictor of outcome compared with iFDG-PET/CT.

**Supplementary Information:**

The online version contains supplementary material available at 10.1007/s00259-021-05353-9.

## Introduction

^18^F-Fluorodeoxyglucose (FDG) positron emission tomography (PET) with integrated computed tomography (FDG-PET/CT) is a well-established approach in the staging, restaging, and response assessment at the end of therapy in diffuse large B cell lymphoma (DLBCL) [1–3]. While the predictive power of end-of-therapy FDG-PET/CT in DLBCL has been confirmed in several retrospective and prospective studies, the predictive value of iFDG-PET/CT remains controversial [4–18].

Theoretically, because metabolic response clearly precedes morphologic change following anti-DLBCL therapy, metabolic changes noted on iFDG-PET/CT should be able to serve as early predictor of anti-tumor response [4–18]. Yet iFDG-PET/CT has shown limited ability in differentiating between favorable and unfavorable outcomes following treatment of DLBCL, most notably with high false positive results. Patients with a negative iFDG-PET/CT maintain long-term remission in 75–85% of cases, but even with a positive iFDG-PET/CT as many as 30–60% of patients also have long-term remission [4–18].

The high and variable percentages of false-positive iFDG-PET/CT scans in the various studies appear to be related to multiple factors, including tumor bulk, timing of PET after chemotherapy, and the outcome methodology, all of which have contributed to lack of dependability of iFDG-PET/CT in DLBCL and hampered its use in clinical practice for making early treatment decisions [4–18]. Perhaps the most important cause of false-positive iFDG-PET/CT is FDG accumulation in post-therapy inflammatory changes [8, 19], which appear to be more pronounced when rituximab is combined with chemotherapy [8]. Standardized interpretation criteria, such as the Deauville criteria, have not resulted in a substantial improvement in the prognostic performance [20].

3′-Deoxy-3′-[^18^F]fluorothymidine (FLT) has been shown to be an excellent imaging biomarker of lymphoma cell proliferation in DLBCL with excellent correlation with Ki-67, the well-established histopathological marker of cellular proliferation [21]. Importantly FLT is less affected by post-therapy inflammatory changes caused by macrophage/monocyte infiltration and therefore likely to be a more tumor-specific tracer compared with FDG, providing the rationale for trials comparing FDG-PET/CT and FLT-PET/CT following chemoimmunotherapy [22].

Our research group hypothesized that iFLT-PET/CT is a superior predictor of outcome compared with iFDG PET/CT in patients with DLBCL treated with rituximab, cyclophosphamide, doxorubicin, vincristine, and prednisone (R-CHOP) or rituximab, etoposide, prednisone, vincristine, cyclophosphamide, and doxorubicin (R-EPOCH).

The aim of this multicenter prospective study is to provide the final results of our prospective multicenter trial following a median follow-up period of 3 years enabling the determination of the predictive values of iFDG-PET/CT and iFLT-PET/CT based on the 3- and 5-year progression-free survival (PFS) rates of patients with positive and negative interim PET scans defined using standardized contemporary evaluation criteria.

## Materials and methods

### Study design

This was a prospective, multi-institutional trial conducted at the University of Texas MD Anderson Cancer Center; University of Nebraska Medical Center; University Hospital of Aachen, Germany; and Stanford University and funded by the National Institutes of Health (grant 1R01CA 152923–01A). This study was approved by the institutional review board at each of the four institutions and is fully compliant with the Health Insurance Portability and Accountability Act. Written informed consent was obtained from all of the subjects.

To be eligible for the trial, patients must have (a) received a new histologic or cytologic diagnosis of de novo DLBCL; (b) been scheduled to receive first-line chemotherapy with R-CHOP or R-EPOCH given every 21 days for six cycles with or without consolidative external radiation therapy; (c) had an Eastern Cooperative Oncology Group performance status of 0–2; and (d) had a minimal life expectancy of 6 months. Exclusion criteria were patients with any history of prior lymphoma, the presence of a second cancer (aside from basal cell carcinoma), and pregnancy.

The International Prognostic Index (IPI) was obtained for each patient. Staging (baseline) FDG PET/CT, conventional imaging, and clinical examination (including bone marrow biopsy) were performed before therapy initiation.

The iFDG-PET/CT and iFLT-PET/CT findings were compared with PFS obtained based on routine follow-up clinical examination, CT, and/or FDG-PET/CT scans. PFS was calculated from the start of R-CHOP or R-EPOCH until progression of DLBCL or last follow-up. No therapy change was made on the basis of iFDG-PET/CT or iFLT-PET/CT unless progression was documented by CT and/or biopsy. Pretherapeutic and end-of-treatment FLT-PET scans were not performed because of restrictions imposed by the funding agency, institutional review boards, and radiation safety committees due to serious concerns regarding excess radiation exposure to the enrolled patients, especially since the primary goal of our study was to compare iFLT-PET with iFDG-PET.

### PET/CT imaging

iFDG-PET/CT and iFLT-PET/CT were both performed 18–24 days after the second cycle of R-CHOP or R-EPOCH. FDG- and FLT-PET/CT studies were performed within a mean of 1.4 ± 1.0 days of each other (range, 1–6 days). Noncontrast-enhanced FDG- and FLT-PET/CT studies were performed 60–70 min after injection of ~370 MBq and 185 MBq of FDG and FLT, respectively, using Discovery PET/CT scanner (GE Healthcare, Waukesha, WI) or Gemini time-of-flight 16 or 64-section PET/CT scanner (Philips Healthcare, Best, the Netherlands), as previously described [22].

### FDG-PET/CT image analysis

All FDG and FLT-PET/CT studies were reviewed by two Nuclear Medicine physicians in consensus, who were blinded to the clinical data and the results of other imaging studies, using MIMvista software 6.7 (MIMvista, Cleveland, OH). Each study was reviewed to determine the index lesion (s) for qualitative and quantitative assessment that best represented the overall disease in each patient.

The primary analysis of interim iFDG-PET/CT studies was based on the Deauville response criteria [20] (adopted in the Lugano criteria [2] and PET Response Criteria in Solid Tumors 1.0 (PERCIST) [23]). To facilitate comparison between the various criteria, iFDG PET/CT findings were classified as either positive or negative after the primary interpretation. For the Deauville criteria [20], which are based on a 5-point scale, studies with a score of 4 or 5 were classified as positive and studies with a score of 1–3 were classified as negative. For PERCIST version 1.0, studies interpreted as showing partial metabolic response (PMR), stable metabolic disease (SMD), or progressive metabolic disease (PMD) were classified as positive while studies interpreted as showing complete metabolic response (CMR) were classified as negative.

For quantitative analysis of iFDG-PET/CT, we used the SUV_max_ of the lesion with the most intense FDG uptake on baseline and iFDG-PET/CT, the percentage change in SUV_max_ between baseline and iFDG-PET/CT, baseline and interim metabolic tumor volume (MTV), baseline and interim total lesion glycolysis (TLG), the percentage change in MTV between baseline and iFDG-PET/CT and the percentage change in TLG between baseline and iFDG-PET/CT. The SUV_max_, lean body mass–corrected SUV peak or SUL_peak_ (used for PERCIST analysis) of whole-body lesions, MTV, and TLG were measured with the aid of the PETedge tool within the MIMvista software. The PETedge tool uses a gradient-based tumor segmentation method with manual adjustment [24].

### iFLT PET/CT image analysis

We defined positive FLT uptake as visually higher than that of the uptake of left atrium (blood pool); therefore, SUV_max_ of abnormal FLT uptake and FLT mean SUV in the left atrium were measured and recorded. For skeletal lesions, we measured the FLT uptake (SUV_max_) at the site of abnormality on the baseline FDG-PET/CT image. Skeletal lesions were then defined as negative for residual active disease if the FLT SUV_max_ of the skeletal lesion was less than or equal to that of the surrounding (immediately adjacent) skeletal structure. A 1-cm-diameter region-of-interest was used for measurement of FLT SUV_max_ of the surrounding skeletal region.

### Statistical considerations

#### Sample size justification

A total of 137 subjects were initially planned to be enrolled in the study. This sample size was based on detecting an expected difference of ≥23% in PFS between the iFLT-PET/CT-positive and iFDG-PET/CT-positive patients with >90% power at the 0.05 level of significance. At the same time, this sample size was sufficient to ensure that iFDG-PET/CT-positive/iFLT-PET/CT-negative patients will not have >20% lower PFS compared with iFDG-PET/CT-negative/iFLT-PET/CT-negative patients, which would be detected with 80% power at the 0.10 level of significance.

We estimated that the maximal 20% PFS difference between the iFDG-PET/CT-positive/iFLT-PET/CT-negative and the iFDG-PET/CT-negative/iFLT-PET/CT-negative patients would translate to <5% lower NPV for iFLT-PET/CT vs. iFDG-PET/CT since the majority of patients (60%) are expected to have negative iPET/CT scans with both tracers with expectedly excellent outcome. Due to this small expected difference, a direct comparison of the NPVs of iFLT-PET/CT and iFDG-PET/CT would have required a very large sample size (> 400 patients).

An interim analysis performed after accrual of 60 patients with 46 evaluable (median follow-up = 13 months) showed a greater than expected, statistically highly significant difference in PFS between the iFLT-PET/CT-positive and iFDG-PET/CT-positive patients (47% difference, 9% PFS for iFLT vs. 56% PFS for iFDG) with negligible difference in NPV between iFDG-PET/CT and iFLT-PET/CT (1% difference, 94% for iFLT vs. 95% for iFDG). It was, therefore, decided to extend accrual to only about 100 patients with confirmed performance of iFDG-PET/CT and iFLT-PET/CT at the same time rather than accruing the originally planned target of 137 subjects. The interim analysis was not part of the initial study design. However, because of limited funding available for this complex study and our initial observation of much higher percentage of negative iFLT-compared with negative iFDG-PET who mostly had negative end-of-treatment FDG-PET, it was estimated that a lower number of patients will suffice to address the research question.

### Statistical analysis

The differences between baseline and iFDG-PET/CT parameters in lesions (SUV_max_, SUL_peak_, TLG, and MTV) and reference organs (left atrium SUV_mean_, liver SUV_mean_, liver SUL and liver SUL standard deviation) were compared by using the Wilcoxon signed-rank test. A post hoc analysis of the incidence of events within 5 years from the start of R-CHOP or R-EPOCH was performed, and receiver operating characteristic (ROC) analysis was used to obtain suitable cutoff points (Youden index) for baseline and interim SUV_max_, MTV, and TLG as well as percentage change in these parameters between baseline and iFDG PET/CT.

PFS analysis was performed using the Kaplan–Meier method for dichotomous variables and compared using the log-rank test. The variables associated with PFS were evaluated with the univariate and multivariate Cox proportional hazards regression model of which the PET scan results after two cycles of R-CHOP or R-EPOCH with other indexes were censored after 3 or 5 years, or at withdrawal of treatment or follow-up. All statistical analyses were performed with Stata 14 (Stata, College Station, TX). Calculated *P*-values were two sided, with *P* < .05 considered to indicate a statistically significant difference.

Four surviving patients were diagnosed with a 2nd malignancy (without any evidence of recurrent DLBCL) in the follow-up period, and were censored as no recurrence at the time of the diagnosis.

## Results

### Patient characteristics

From November 2011 to September 2015, 122 patients were eligible for this study. Thirty patients were excluded from this study due to withdrawal of consent and/or inability to continue treatment without evidence of disease progression (*n* = 18) or FLT production failure (*n* = 12). Ninety-two patients completed FDG and FLT imaging and are the subject of this analysis. The characteristics of the enrolled patients are shown in Table 1. Outcome data revealed 21 of 92 patients had persistent/relapsed disease within 5 years from the initiation of chemotherapy. Nine patients died: 7 from progression of DLBCL, one from sepsis following disease progression, and one from poorly differentiated squamous cell carcinoma with no evidence of DLBCL progression at the time of death.

**Table 1:** Characteristics of the 92 eligible patients

Characteristic	Datum
Age (y)^a^	
All patients	59.0 ± 15.1 (21–88)
Male patients	59.8 ± 15.4 (22–88)
Female patients	57.3 ± 14.2 (21–83)
Gender	
Male	54 (58.7)
Female	38 (41.3)
Stage	
I	8 (8.7)
II	23 (23.9)
III	22 (23.9)
IV	39 (42.4)
International Prognosis Index score	
0	8 (8.7)
1	26 (28.3)
2	26 (28.3)
3	15 (16.3)
4	9 (9.8)
5	1 (1.1)
Undefined	7 (7.6)
Bone marrow biopsy	
Positive	13 (14.1)
Negative	78 (84.8)
Not performed	1 (1.1)

Data are numbers of patients, with percentages in parentheses

^a^Data are means ± standard deviations, with ranges in parentheses

### PET/CT imaging parameters at baseline and interim response assessment

Table 2 shows the PET/CT imaging parameters of baseline and iFDG-PET/CT as well as iFLT-PET/CT.

**Table 2:** FDG and FLT PET/CT imaging parameters at baseline and interim scan

Index	Baseline PET	Interim PET	*P* value
FDG PET/CT			
Lesion measurements			
SUV_max_	24.6 ± 11.2 (6.5–73.6)	4.3 ± 4.9 (0–33.4)	< 0.001
MTV (ml)	594.9 ± 727.5 (3.4–3025.1)	108.0 ± 216.7 (0–1434.8)	< 0.001
TLG (g·10^−3^)	5405.3 ± 6955.3 (17.0–26,979.8)	231.7 ± 453.5 (0–3079.6)	< 0.001
SUL peak	13.8 ± 6.0 (3.1–36.0)	2.1 ± 2.1 (0–10.6)	< 0.001
Reference organs			
Left atrium SUV_mean_	1.7 ± 0.5 (0.7–3.1)	1.9 ± 0.4 (0.8–2.9)	0.09
Liver SUV_mean_	2.1 ± 0.5 (1.1–3.6)	2.3 ± 0.5 (0.5–4.4)	0.002
Liver SUL	1.5 ± 0.3 (0.8–2.6)	1.7 ± 0.3 (0.6–2.4)	< 0.001
Liver SUL standard deviation	0.16 ± 0.06 (0.07–0.38)	0.16 ± 0.05 (0.09–0.33)	0.57
iFLT-PET/CT			
Lesion measurements			
SUV_max_ of positive lesions^a^		6.0 ± 3.2 (2.3–18.2)	
Reference organs			
Left atrium SUV_mean_		0.9 ± 0.4 (0.4–1.9)	

Data are means ± standard deviations, with ranges in parentheses. ^a^FLT SUV_max_ values for soft-tissue lesions and lymph nodes are reported only for visually positive lesions; data in negative lesions are not reported because most could not be visually seen to enable the drawing of an adequate region of interest

At iFDG-PET/CT, the SUV_max_, TLG, MTV, and SUL_peak_ were significantly decreased after two cycles of treatment compared with baseline. The left atrium and liver uptake at iFDG-PET/CT was slightly increased from those at baseline.

The mean SUV_max_ of FLT uptake in visually positive lesions was 6.0 ± 3.2 in 37 lesions (35 soft-tissue masses or lymph nodes and two skeletal lesions) from 25 patients. The mean SUV_max_ of FLT uptake in visually positive lesions was 5.9 ± 3.3 in extra skeletal masses and 7.7 in the 2 skeletal lesions. Skeletal lesions at interim FLT PET/CT typically showed photopenic or background FLT uptake at each disease site regarded as negative.

### Concordance/discordance of response assessment between Deauville and iFLT-PET/CT vs. patient outcome

Concordance/discordance of interim response assessment between Deauville and iFLT-PET/CT is shown in Table 3.

**Table 3:** Concordance/discordance of response assessment between Deauville and iFLT-PET/CT

Deauville score	iFLT-PET/CT assessment		Total
	Positive	Negative	
1	0	26	26
2	1	11	12
3	4	11	15
4	12	15	27
5	8	4	12
Total	25	67	92

**Fig. 1:**
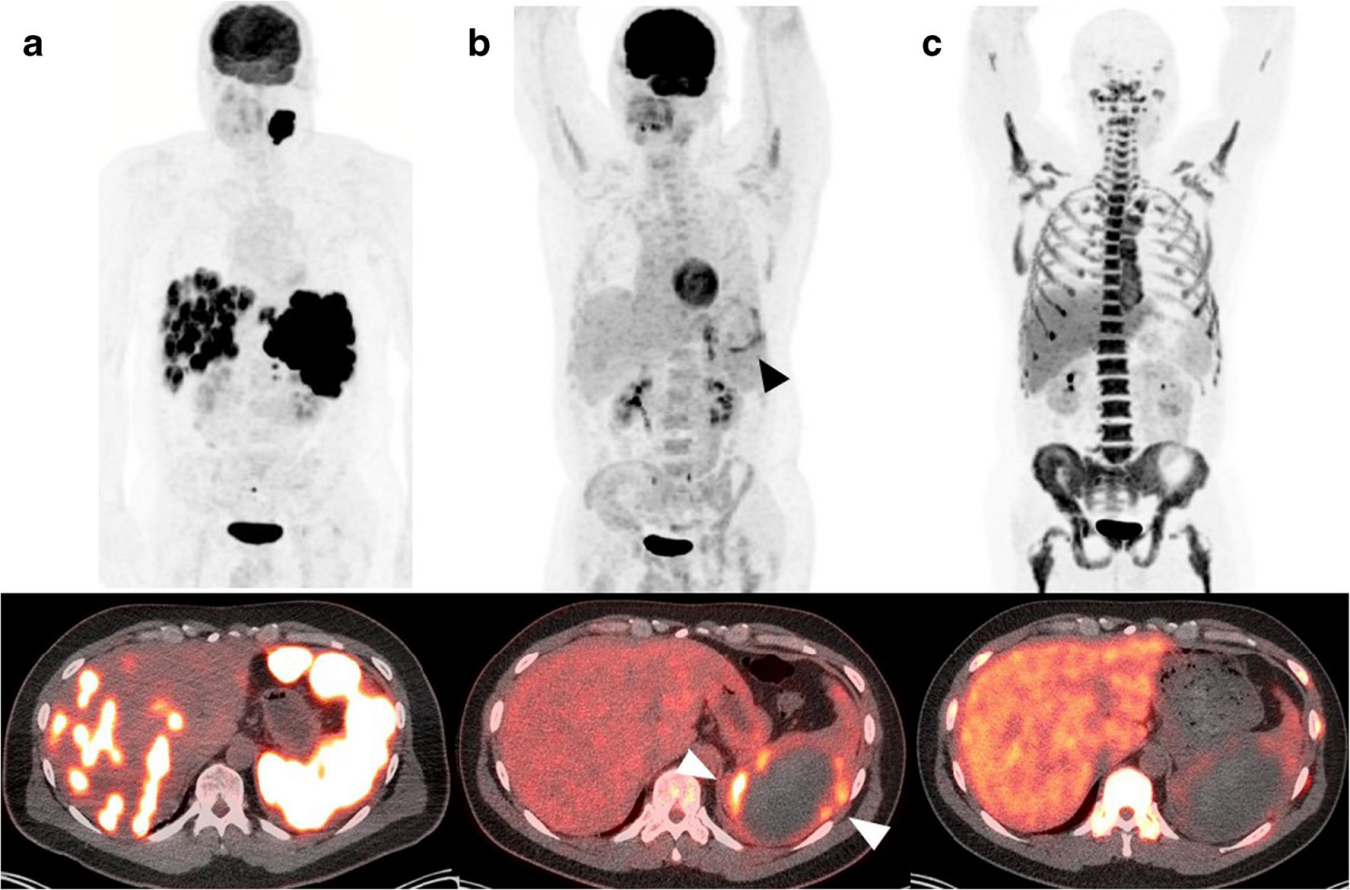
Images in 73-year-old man. **a** Baseline FDG PET maximum intensity projection (upper row) and axial PET/CT fusion image (lower row) show intense FDG uptake in the liver and spleen. **b** Interim FDG PET maximum intensity projection (upper row) and axial PET/CT fusion image (lower row) show decreased activity in the liver and spleen, but the lesion remained positive in spleen (arrowhead) due to an area with visually higher than liver FDG uptake in the spleen. **c** Interim FLT PET image (upper row) and axial PET/CT fusion image (lower row) show no area higher than surrounding area in liver and photopenic tracer uptake in spleen lesion, which was considered negative for disease. This patient remains without evidence of recurrence for 3 years and 2 months from initiation of treatment

**Fig. 2:**
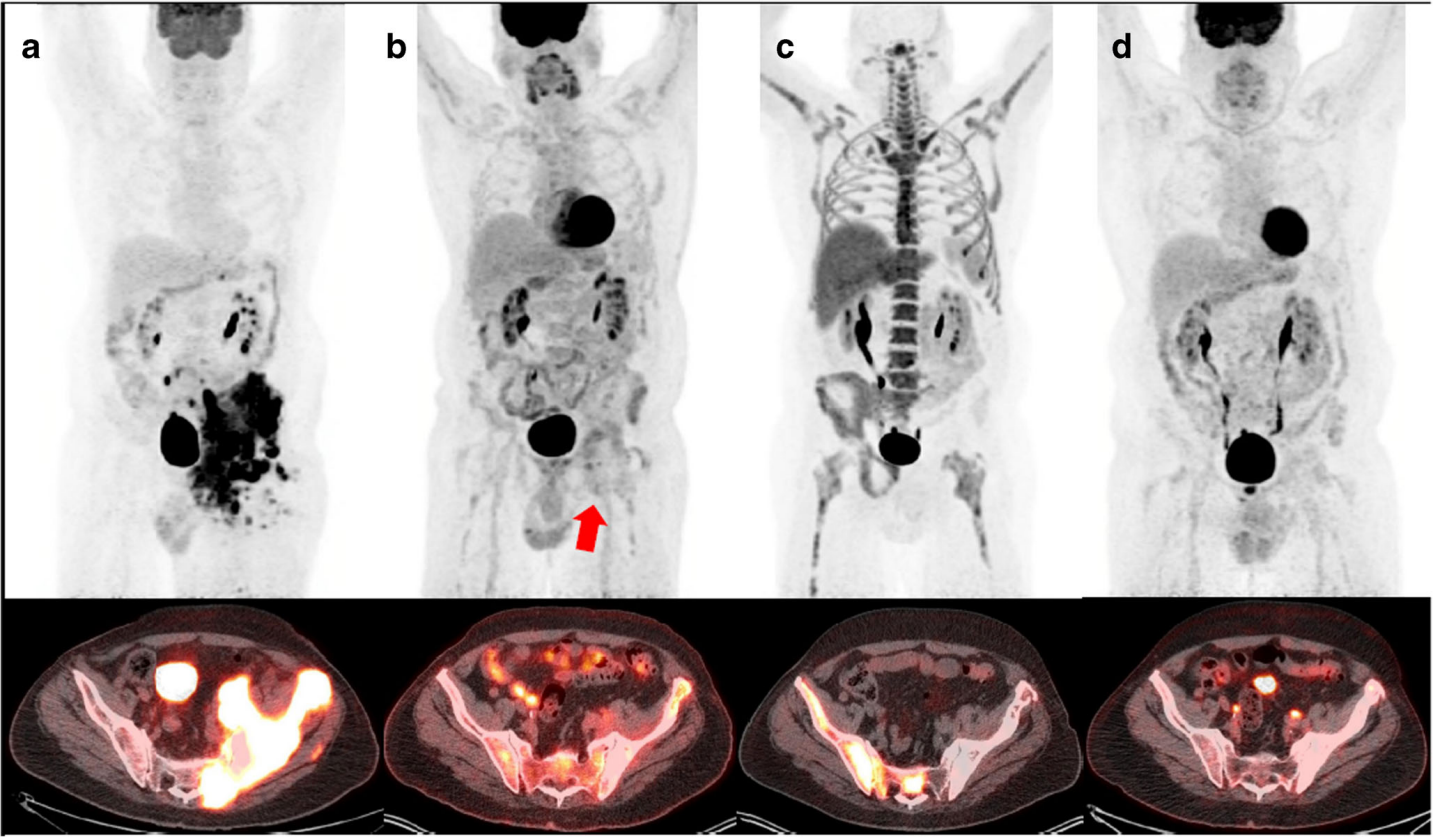
Images in 46-year-old man. **a** Baseline FDG PET maximum intensity projection (upper row) and axial PET/CT fusion image (lower row) show intense FDG uptake in a bulky mass within the left pelvis. **b** Interim FDG PET maximum intensity projection (upper row) and axial PET/CT fusion image (lower row) show decreased activity, but the lesion remained positive (arrow) due to an area with visually higher than liver FDG uptake. **c** Interim FLT PET image (upper row) and axial PET/CT fusion image (lower row) show photopenic tracer uptake in this lesion, which was considered negative for disease. **d** End-of-treatment FDG PET maximum intensity projection shows (upper row) and axial PET/CT fusion image (lower row) substantial reduction in FDG activity which is visually same with mediastinal FDG uptake. This patient remains without evidence of recurrence for 3 years from initiation of treatment

Only 25 of 92 patients had measurable iFLT uptake compared to 65 of 92 patients with measurable iFDG uptake. Using the Deauville criteria in comparison with iFLT-PET/CT, 68 patients (73.9%) had concordant response classification (Deauville scores 1–3 and FLT negative or Deauville scores 4 and 5 and FLT positive) while 24 patients had discordant response classification. Figures 1 and 2 show representative examples of discordance between the Deauville criteria and iFLT-PET/CT. The most pronounced discordance was observed in the patients with Deauville scores of 4 and 5 (positive by Deauville criteria), of whom almost half (19/39 or 48.7%) were iFLT-PET/CT negative. Seventeen of these 19 patients (89.5%) remain progression-free at 6.7 to 60.0 months (median, 37.3 months) of follow-up; the 3- and 5- year PFS rates in this group are 89.5 and 89.5%, respectively. In contrast, 10 of the 20 patients (50.0%) with concordant response classification of persistent disease (Deauville scores 4 and 5 and FLT positive) progressed at a median of 4.2 months from the start of R-CHOP or R-EPOCH with the other 10 patients remaining progression-free at 22.1 to 60.0 months (median, 43.8 months) of follow-up.

A relatively minor discordance was also observed in the patients with Deauville scores of 1–3 (negative by Deauville criteria), of whom 9.4% (5/53) patients were iFLT-PET/CT positive. One of those 5 patients remains progression-free at 51.9 months of follow-up, and 4 patients progressed at a median of 15.0 months post-therapy.

Finally, 43 of the 48 patients (89.6%) with concordant classification of complete response (Deauville scores 1–3 and FLT negative) remain progression-free at 4.8 to 60.0 months (median, 36.2 months) of follow-up.

Similar results were obtained comparing PERCIST with iFLT-PET/CT (see text and Table S1 in the Online Supplement).

### Progression-free survival according to interpretation criteria

Median follow-up for surviving patients was 36 months (3–60 months). The 3- and 5-year PFS rates for the 92 patients were 77.9% (95%CI: 67–85%) and 72.4% (95%CI: 59–82%), respectively.

Table 4 lists the 3- and 5-year PFS rates in the most relevant response categories based on iFLT-PET/CT and Deauville criteria.

**Table 4:** The 3- and 5-year PFS rates in the various response categories based on iFLT-PET/CT and Deauville criteria

	3-year PFS rates (%), 95% CI	5-year PFS rates (%), 95% CI
iFLT-PET/CT (+) n = 25	47.7 [27–65]	40.9 [20–60]
iFLT-PET/CT (−) *n* = 67	89.8 [78–95]	86.0 [70–93]
Deauville (+) *n* = 39	70.5 [53–83]	63.4 [42–79]
Deauville (−) *n* = 53	83.8 [70–92]	79.2 [62–89]
iFLT-PET/CT (−)/Deauville (+) *n* = 19	87.5 [58–97]	87.5 [58–97]
iFLT-PET/CT (−)/Deauville (−) *n* = 48	91.3 [78–97]	86.0 [67–94]

(+): positive, (−): negative

Of the 67 iFLT-PET/CT-negative patients, 7 (10.4%) progressed at a median of 14.1 months (range, 3.6 to 40.8 months). In contrast, 14 of the 25 (56.0%) iFLT-PET/CT-positive patients progressed at a median of 7.8 months (range, 2.6 to 42.2 months) (*P* < .0001). Of the 53 Deauville-negative patients, 9 (17.0%) progressed at a median of 14.1 months (range, 3.6 to 40.8 months) whereas 12 of the 39 patients (30.8%) who were Deauville-positive progressed at a median of 5.6 months (range, 2.6 to 42.2 months) (*P* = .11). The 3- and 5-year PFS rates of the 19 patients who were iFLT-PET/CT-negative but Deauville-positive were 87.5% and 87.5%, respectively, compared with 91.3% and 86.0%, respectively, for the 48 patients who were negative by both iFLT-PET/CT and Deauville.

A similar pattern was seen using PERCIST (see text and Table S2 in the Online Supplement).

Univariate and multivariate analyses to identify predictors for 3- and 5-year PFS are shown in Tables S3 and S4 in the Online Supplement. In univariate analysis, iFLT-PET/CT (HR 9.67, 95%CI: 3.48–26.89, *P* < .0001), PERCIST (HR 2.57, 95%CI: 1.04–6.33, *P* = .04), and interim TLG (HR 4.77, 95%CI: 1.10–20.64, *P* = .04) were significant predictors of 3-year PFS while iFLT-PET/CT (HR 6.71, 95%CI: 2.70–16.67, *P* < .0001), interim SUV_max_ (HR 2.96, 95%CI: 1.08–8.09, *P* = .034), and interim TLG (HR 3.50, 95%CI: 1.03–11.9, *P* = .045) were significant predictors of 5-year PFS. Multivariate analysis showed that iFLT- PET/CT was the only significant independent predictor of 3-year PFS (HR 8.13, 95%CI: 2.55–25.91, *P* < .0001) and 5-year PFS (HR 5.54, 95%CI: 1.97–15.60, *P* = .001).

**Fig. 3:**
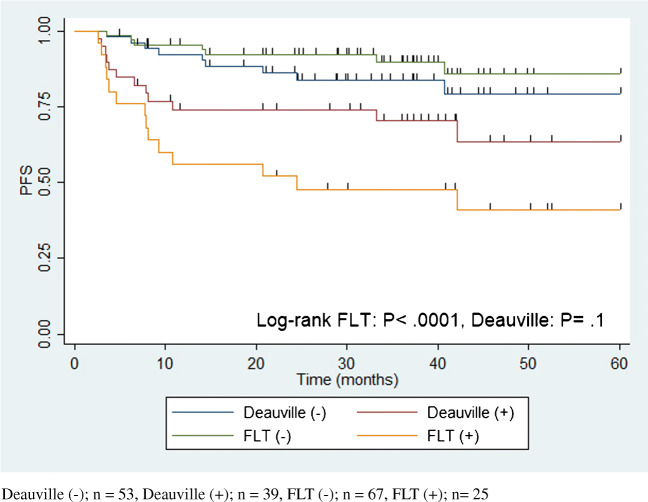
PFS as stratified by Interim FLT-PET/CT and Deauville criteria. Deauville (−); *n* = 53, Deauville (+); *n* = 39, FLT (−); *n* = 67, FLT (+); *n* = 25

Figure 3 shows the Kaplan–Meier plots of PFS for the patients with negative iFLT-PET/CT, positive iFLT-PET/CT, and negative and positive Deauville classifications. PFS was significantly shorter in patients with positive iFLT-PET/CT compared with those with negative iFLT-PET/CT (*P* < .0001) with no significant difference in PFS between the Deauville-positive and Deauville-negative patients (*P* = .1).

**Fig. 4:**
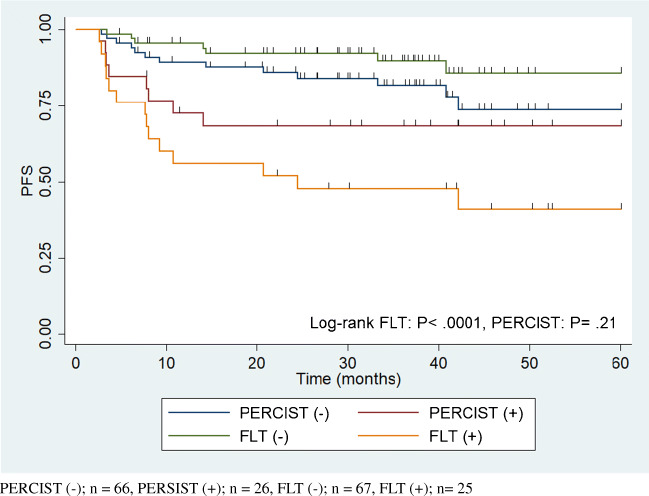
PFS as stratified by Interim FLT-PET/CT and PERCIST. PERCIST (−); *n* = 66, PERSIST (+); *n* = 26, FLT (−); *n* = 67, FLT (+); *n* = 25

Figure 4 shows the Kaplan–Meier plots of PFS for the patients with negative iFLT-PET/CT, positive iFLT-PET/CT, and negative and positive PERCIST. Here again, PFS was significantly shorter in patients with positive iFLT-PET/CT compared with those with negative iFLT-PET/CT (*P* < .0001) with no difference in PFS between the PERCIST-positive and PERCIST-negative patients (*P* = .21).

When we performed a subanalysis focusing exclusively on the 78 patients who received R-CHOP, similar findings were seen with highly significant difference between the performance of iFLT and iFDG-PET. In univariate analysis, iFLT-PET/CT (HR 4.56, 95%CI: 1.77–11.78, *P* = .002), baseline MTV (HR 4.95, 95%CI: 1.54–15.95, *P* = .007), and interim TLG (HR 4.77, 95%CI: 1.10–20.64, *P* = .04) were significant predictors of 3-year PFS while iFLT-PET/CT (HR 5.56, 95%CI: 2.19–14.07, *P* < .0001) and baseline MTV (HR 4.52, 95%CI: 1.40–14.57, *P* = .012) were significant predictors of 5-year PFS. Multivariate analysis showed that iFLT- PET/CT was the only significant independent predictor of 3-year PFS (HR 4.83 95%CI: 1.62–14.35, *P* = .005) and 5-year PFS (HR 5.53 95%CI: 1.99–14.8, *P* = .001). PFS was significantly shorter in patients with positive iFLT-PET/CT compared with those with negative iFLT-PET/CT (*P* < .0001) with no significant difference in PFS between the Deauville-positive and Deauville-negative patients (*P* = .46) and in PFS between the PERCIST-positive and PERCIST-negative patients (*P* = .52).

## Discussion

The main purpose of this prospective head-to-head comparison was to determine whether iFLT-PET/CT is a superior predictor of PFS compared with iFDG-PET/CT in patients with DLBCL treated with 6 cycles of R-CHOP or R-EPOCH given every 21 days.

The important finding of our study is that iFLT-PET/CT showed a highly significant difference in PFS between patients with negative and positive scans whereas there was no significant difference in PFS between iFDG-PET/CT-negative and iFDG-PET/CT-positive scans regardless of whether the Deauville or PERCIST criteria were used to define negativity/positivity.

There was a substantially lower proportion of patients with a positive scan using iFLT-PET/CT compared with iFDG-PET/CT by the Deauville criteria (27% vs. 42%) with increased iFLT-PET/CT specificity/PPV and preserved sensitivity/NPV. Most notably, 17/19 iFDG-PET/CT-positive/iFLT-PET/CT-negative patients had a favorable outcome with long PFS (median = 37.3 months) that was similar to those who were iFDG-PET/CT-negative. This suggests that iFLT-PET/CT identifies a distinct subgroup within the iFDG-PET/CT-positive patients in which the increased FDG-PET uptake is not due to residual tumor but rather to post-therapy inflammatory changes. When we followed the 3- and 5-year outcomes of the high percentage of patients with negative FLT-PET/CT (73%), we found that their PFS were as high as those of iFDG-PET/CT-negative patients. The preserved NPV of iFLT-PET/CT is critical for consideration of this imaging for response assessment of DLBCL.

The poor predictive ability of iFDG-PET/CT using the Deauville criteria found in our study is similar to what has been reported in two recent prospective studies [25, 26]. In the Cancer and Leukemia Group B investigation, 35% of 158 evaluable DLBCL patients who underwent FDG-PET/CT after 2 cycles of chemotherapy were positive by Deauville criteria but these criteria failed to predict outcome after a median follow-up of 5 years [25]. ΔSUV on iFDG-PET/CT predicted overall survival (OS) but not PFS, although the low number of events limited the statistical analysis. In the UK National Cancer Research Institute study, 189 DLBCL patients given R-CHOP had baseline and post-cycle-2 FDG-PET [26]. After a median follow-up of 5.4 years, patients with Deauville scores of 1–3 at iFDG-PET/CT had higher end-of-treatment complete and overall response rate; however, only a Deauville score of 5 was associated with inferior PFS and OS [26]. This led the investigators to conclude that post-cycle-2 FDG-PET/CT has limited value in identifying patients with poor outcome.

Despite its clear superiority to iFDG-PET/CT, our data also indicate that the specificity of iFLT-PET/CT is imperfect. However, the fraction of “false-positive” iFLT-PET/CT scans in the overall patient population is small (11/92 or 12%) compared to 29.3% (27/92) with iFDG-PET/CT using Deauville. The false-positive FLT scans are possibly related to proliferation of potential “resident” tumor macrophages, which may be present in the DLBCL microenvironment similar to other lymphoma types, such as T cell/histiocyte-rich large B cell lymphoma [27–30].

Due to the imperfect specificity of iFLT-PET/CT, biopsy of FLT-positive lesions still needs to be performed (unless there is other compelling evidence of disease) before contemplating a change in treatment. It is important to emphasize, however, that biopsy would then be required in only about one-fourth of all patients (27%) of whom more than half are likely to have evidence of disease at biopsy. Thus, unnecessary biopsy would be done in only ~12% of all patients. On the other hand, given their excellent prognosis, patients who are iFLT-PET/CT-negative (~the remaining three-fourths) may be the target for de-escalation strategies in the context of clinical trials. For example, it might be interesting to investigate the utility of using negative iFLT-PET in patients with DLBCL to determine whether 4 rather than 6 cycles of R-CHOP may be given with maintained efficacy and reduced toxicity.

A prior study by Schöder et al. also investigated the predictive value of iFLT-PET/CT compared with iFDG-PET/CT in aggressive B cell lymphomas [31]. In that study iFLT-PET/CT was performed after either 1 or 2 cycles of R-CHOP-14 while iFDG-PET/CT was performed after four R-CHOP-14 cycles making it difficult to obtain a direct comparison of the predictive values of both imaging tools. Moreover, due to the relatively small sample size, the investigators could not determine whether post-cycle 1 or 2 iFLT-PET/CT is more predictive of outcome. Nevertheless, similar to our study, a high percentage (78%) of negative iFLT-PET/CT scans were found after 2 cycles of R-CHOP-14 and iFLT-PET/CT was predictive of both PFS and OS.

Because of the relatively high physiological bone marrow activity, post-therapy assessment of skeletal lesions is somewhat challenging with FLT-PET/CT. As shown in Fig. 2, FLT tends to show less uptake in treated skeletal lesions compared with surrounding normal bone marrow. This is likely related to the fact that marrow-replacing tumor cells at the treated skeletal site become necrotic and that any subsequent post-therapy inflammatory changes do not concentrate much FLT, at least not to the extent of FDG uptake. As the bone marrow at the previously involved skeletal site repopulates, will the FLT uptake begin to normalize.

A relative limitation of our study pertains to treatment heterogeneity as patients received R-CHOP or R-EPOCH for treatment. However, R-EPOCH was only given to 14 patients (15%) and recent reports have shown that dose-adjusted R-EPOCH is equivalent to R-CHOP with respect to PFS and OS [32]. All patients who received R-EPOCH or R-CHOP were imaged with both FLT and FDG, and the head-to-head comparison of both tracers was accomplished using the same treatment. Furthermore, a subanalysis focusing on the patients who received R-CHOP also showed significant difference between the performance of iFLT and iFDG-PET/CT.

Another limitation of our study is that FLT production failed in about 10% of our patients. In the course of the trial, it became clear that reliable FLT production with virtually 0% failure rate is achieved when a fully automated FLT synthesis, for example, using nucleophilic fluorination catalyzed by protic solvent, is implemented [33]. We believe that institutional and potentially commercial radiopharmacies will be capable of FLT production with minimal production fails when following standardized protocols using fully automated FLT synthesis. It is also noteworthy that, already in 2009, the U.S. Food and Drug Administration approved the Society of Nuclear Medicine (SNM) centralized multicenter investigational new drug application for FLT enabling FLT imaging in large therapeutic clinical trials and representing an important step towards final approval of this tracer for routine use.

Future research should investigate the role of FLT-PET at the end of treatment given the 20–30% false-positive FDG-PET findings in this setting. A comparison of end-of-treatment FLT-PET with end-of-treatment FDG-PET should be conducted in a separate study to determine whether the former is significantly superior. Furthermore, the utility of the change in tumoral FLT uptake between baseline and various post-therapy timepoints should be assessed to determine whether this change provides a more accurate measure than absolute FLT uptake at a particular timepoint.

Finally, it is interesting to speculate whether FLT could become the new standard in response monitoring of aggressive lymphomas, at least in the interim setting. Based on our data, using iFLT rather than iFDG in response monitoring of DLBCL would reduce the number of needed biopsies by 36% (from 42 to 27%). iFLT-negative/iFDG-positive patients will do just as well as those negative with both tracers with a 3-year PFS of almost 90%, and the lower 3-year PFS rate in iFLT-positive compared with iFDG-positive patients (47.7% vs. 70.5%) suggests greater iFLT specificity with greater likelihood of a positive biopsy. As for the FLT availability, we believe that once its clinical utility has been clearly established in a certain setting, all potential obstacles will be overcome to make it widely available using standardized production protocols.

## Conclusion

This multicenter head-to-head comparison shows that iFLT-PET/CT is superior to iFDG-PET/CT using both quantitative assessment and therapeutic assessment criteria in predicting PFS of DLBCL given R-CHOP or R-EPOCH and provides the rationale for using FLT-PET/CT in lieu of or in addition to FDG-PET/CT for interim response assessment of DLBCL with implications for improved patient management.

## Supplementary Information

ESM 1 (DOCX 36.7 kb)

ESM 2 (DOCX 44.1 kb)

## Data Availability

Raw data were generated at the University of Texas MD Anderson Cancer Center, University of Nebraska Medical Center, University Hospital of Aachen, Germany, and Stanford University. Derived data supporting the findings of this study are available from AQ or MJ on request.

## Abbreviations

CMR: Complete metabolic response
DLBCL: Diffuse large B cell lymphoma
FDG: Fluorine 18 fluorodeoxyglucose
FLT: Fluorine 18 fluorothymidine
IHP: International Harmonization Project
IWG: International Working Group
MTV: Metabolic tumor volume
NPV: Negative predictive value
PERCIST: PET Response Criteria in Solid Tumors
PMD: Progressive metabolic disease
PMR: Partial metabolic response
PPV: Positive predictive value
PFS: Progression-free survival
R-CHOP: Rituximab, cyclophosphamide, doxorubicin, vincristine, and prednisone
R-EPOCH: Rituximab, etoposide, prednisone, vincristine, cyclophosphamide, and doxorubicin
SMD: Stable metabolic disease
SUL: Lean body mass–corrected SUV
SUV: Standardized uptake value
SUV_max_: Maximum SUV
SUV_mean_: Mean SUV
TLG: Total lesion glycolysis
