# Supplementary material for: 18F-Fluorothymidine PET is an early and superior predictor of progression-free survival following chemoimmunotherapy of diffuse large B cell lymphoma: a multicenter study
Source: Eur J Nucl Med Mol Imaging. 2021 Apr 28;48(9):2883–93. doi: 10.1007/s00259-021-05353-9 (PMC8263539; doi:10.1007/s00259-021-05353-9)
Supplement: Supplementary file 1 — (DOCX 36.7 kb) [file 259_2021_5353_MOESM1_ESM.docx]

**Online Supplement**

**^18^F-Fluorothymidine PET is an Early and Superior Predictor of Progression-Free Survival Following Chemoimmunotherapy of Diffuse Large B Cell Lymphoma: A Multicenter Study**

**Authors**

Ryogo Minamimoto,^1,2^ MD, PhD, Luis Fayad, ^3^ MD, Julie Vose,^4^ MD, MBA, Jane Meza,^5^ PhD, Ranjana Advani, ^6^ MD, Jordan Hankins, ^7^ MD, Felix Mottaghy, ^8^ MD, PhD, Homer Macapinlac,^9^ MD, Alexarder Heinzel, ^8^ MD, Malik E. Juweid, ^10^ MD*,^¥^ Andrew Quon, ^1,11^ MD*

**Concordance/discordance of response assessment between PERCIST and iFLT-PET/C vs. patient outcome**

Concordance/discordance of interim response assessment between PERCIST and iFLT-PET/CT is shown in Table 1S.

When using PERCIST in comparison with iFLT-PET/CT, 71 patients (77.2%) had concordant response classification (CMR and FLT negative or PMR, SMD and PMD and FLT positive) while 21 patients had discordant response classification. The pattern of concordance/discordance using PERCIST was similar to that observed with Deauville criteria. Eleven of the 26 patients (42.3%) with positive PERCIST were iFLT-PET/CT negative with ~ 91% (10/11) remaining progression-free at 7.8 to 60.0 months (median, 38.2 months) of follow-up; the estimated 3- and 5- year PFS rates in this group is 88.9% and 88.9% respectively. On the other hand, 7 of the 15 patients (47%) with concordant response classification of persistent disease progressed at a median of 3.8 months from the start of R-CHOP or R-EPOCH, 8 remained progression-free at 22.1-60.0 months (median, 43.8 months) of follow-up. Here again, only a relatively small fraction of patients who were PERCIST negative were iFLT-PET/CT positive (10/66, 12.1%). Three of those 10 patients remain progression-free at 40.8 to 60.0 months (median, 51.9 months) of follow-up while 7 progressed at a median of 9.3 months posttherapy. As might be expected, 50 Of the 56 patients (89.3%) with concordant classification of complete response remain progression-free at 4.8 to 60.0 months (median, 36.4 months) of follow-up.

**Progression-free survival according to interpretation criteria**

Table 2S lists the 3- and 5-year PFS rates in the most relevant response categories based on iFLT-PET/CT and PERCIST criteria.

Of the 66 PERCIST-negative patients, 13 (19.7%) progressed at a median of 9.3 months (range, 2.9 to 42.2 months) whereas 8 of the 26 (30.8%) PERCIST-positive patients progressed at a median of 5.8 months (range, 2.6 to 14.1 months) (P = 0.22). The estimated 3- and 5-year PFS rates of the 11 patients who were iFLT-PET/CT-negative but PERCIST-positive were 88.9% and 88.9%, respectively compared with 89.7% and 84.7%, respectively for the 56 patients who were negative by both iFLT-PET/CT and PERCIST.
