# Supplementary material for: 18F-Fluorothymidine PET is an early and superior predictor of progression-free survival following chemoimmunotherapy of diffuse large B cell lymphoma: a multicenter study
Source: Eur J Nucl Med Mol Imaging. 2021 Apr 28;48(9):2883–93. doi: 10.1007/s00259-021-05353-9 (PMC8263539; doi:10.1007/s00259-021-05353-9)
Supplement: Supplementary file 2 — (DOCX 44.1 kb) [file 259_2021_5353_MOESM2_ESM.docx]

**Supplemental Tables**

**^18^F-Fluorothymidine PET is an Early and Superior Predictor of Progression-Free Survival Following Chemoimmunotherapy of Diffuse Large B Cell Lymphoma: A Multicenter Study**

**Authors**

Ryogo Minamimoto,^1,2^ MD, PhD, Luis Fayad, ^3^ MD, Julie Vose,^4^ MD, MBA, Jane Meza,^5^ PhD, Ranjana Advani, ^6^ MD, Jordan Hankins, ^7^ MD, Felix Mottaghy, ^8^ MD, PhD, Homer Macapinlac,^9^ MD, Alexarder Heinzel, ^8^ MD, Malik E. Juweid, ^10^ MD*,^¥^ Andrew Quon, ^1,11^ MD*

Table 1S. Concordance/Discordance of Response assessment between

PERCIST and iFLT-PET/CT

|  | iFLT-PET/CT assessment | |  |
| --- | --- | --- | --- |
| PERCIST criteria | Positive | Negative | Total |
| CMR | 10 | 56 | 66 |
| PMR | 9 | 9 | 18 |
| SMD | 3 | 0 | 3 |
| PMD | 3 | 2 | 5 |
| Total | 25 | 67 | 92 |

Table 2S. The 3- and 5-year PFS rates in the various response categories based on

iFLT-PET/CT and PERCIST criteria

|  | 3-year PFS rates (%), 95% CI | 5-year PFS rates (%), 95% CI |
| --- | --- | --- |
| iFLT-PET/CT (+) n = 25 | 47.7 [27-65] | 40.9 [20-60] |
| iFLT-PET/CT (-) n = 67 | 89.8 [78-95] | 86.0 [70-93] |
| PERCIST (+) n = 26 | 68.3 [46-83] | 68.3 [46-83] |
| PERCIST (-) n = 66 | 81.6 [69-89] | 73.6 [57-85] |
| iFLT-PET/CT (-) / PERCIST (+) n = 11 | 88.9 [43-98] | 88.9 [43-98] |
| iFLT-PET/CT (-) / PERCIST (-) n = 56 | 89.7 [76-96] | 84.7 [66-93] |

(+) : positive, (-) : negative

Table 3S. Univariate Analysis for PFS at 3 years and 5 years

| Index | | Hazzard ratio (3-years) | P value | Hazzard ratio (5-years) | P value |
| --- | --- | --- | --- | --- | --- |
| iFLT PET/CT (positive vs negative) | | 9.67 [3.48-26.89] | < 0.0001 | 6.71 [2.70-16.67] | < 0.0001 |
| Clinical stage III or IV vs I or II | | 2.75 [0.80-9.44] | 0.11 | 3.00 [0.88-10.21] | 0.08 |
| International Prognosis Index score >2 | | 1.20 [0.45-3.21] | 0.71 | 1.36 [0.54-3.46] | 0.52 |
| PERCIST (CMR vs PMR or SMD or PMD) | | 2.57 [1.04-6.33] | 0.04 | 1.74 [0.72-4.20] | 0.22 |
| Deauville (1 or 2 or 3 vs 4 or 5) | | 2.10 [0.85-5.23] | 0.10 | 2.03 [0.86-4.82] | 0.11 |
| SUV_max_ | Baseline (cutoff, 22.4 , 0.57) | 1.53 [0.59-3.95] | 0.38 | 1.60 [0.58-4.46] | 0.37 |
|  | Interim (cutoff, 4.2, AUC, 0.65) | 2.55 [0.92-7.08] | 0.07 | 2.96 [1.08-8.09] | 0.034 |
|  | % change (cutoff, 80.0%; AUC, 0.63) | 0.79 [0.28-2.22] | 0.66 | 0.86 [0.33-2.23] | 0.75 |
| MTV | Baseline (cutoff, 298 mL; AUC, 0.61) | 2.62 [0.86-7.96] | 0.07 | 2.90 [0.96-8.76] | 0.06 |
|  | Interim (cutoff, 109 mL; AUC, 0.59) | 2.84 [0.83-9.75] | 0.10 | 2.38 [0.80-7.08] | 0.12 |
|  | % change (cutoff, 83.7%; AUC, 0.53) | 1.29 [0.50-3.34] | 0.60 | 0.85 [0.35-2.05] | 0.72 |
| TLG | Baseline (cutoff, 4087 g·10^-3^; AUC, 0.56) | 2.77 [0.64-12.07] | 0.17 | 3.02 [0.70-13.09] | 0.14 |
|  | Interim (cutoff, 248 g·10^-3^; AUC, 0.60) | 4.77 [1.10-20.64] | 0.04 | 3.50 [1.03-11.9] | 0.045 |
|  | % change (cutoff, 96.3%; AUC, 0.53) | 1.52 [0.59-3.92] | 0.39 | 0.99 [0.41-2.37] | 0.97 |

95%CI is in parentheses.

Table 4S. Multivariate Analysis for PFS at 3 years and 5 years

| Index | Hazzard ratio | P value |
| --- | --- | --- |
| PFS at 3-years |  |  |
| iFLT PET/CT | 8.13 [2.55-25.91] | < 0.0001 |
| PERCIST | 0.93 [0.35-2.47] | 0.89 |
| TLG: Interim (cutoff, 248 g·10^-3^; AUC, 0.60) | 1.85 [0.37-9.30] | 0.45 |
| PFS at 5-years |  |  |
| iFLT PET/CT | 5.54 [1.97-15.60] | 0.001 |
| SUV_max_ Interim (cutoff, 4.2, AUC, 0.65) | 1.11 [0.33-3.72] | 0.86 |
| TLG: Interim (cutoff, 248 g·10^-3^; AUC, 0.60) | 1.53 [0.36-6.53] | 0.57 |

95%CI is in parenthesis
